# Microstructural white matter alterations and cognitive impairment in anti-NMDAR encephalitis: evidence from T1w/T2w ratio analysis

**DOI:** 10.3389/fimmu.2026.1796544

**Published:** 2026-04-29

**Authors:** Jinyu Tao, Shuang Ding, Yayun Xiang, Qiyuan Zhu, Zhuowei Shi, Run Du, Yongmei Li, Chun Zeng

**Affiliations:** 1 Department of Radiology, The First Affiliated Hospital of Chongqing Medical University, Chongqing, China; 2 Department of Radiology, Children’s Hospital of Chongqing Medical University; National Clinical Research Center for Child Health and Disorders, Ministry of Education; Key Laboratory of Child Development and Disorders, Chongqing Key Laboratory of Child Neurodevelopment and Cognitive Disorders, Chongqing, China

**Keywords:** anti-NMDAR encephalitis, cognitive impairment, magnetic resonance imaging, normal-appearing white matter (NAWM), T1w/T2w ratio, white matter

## Abstract

**Objective:**

Anti-N-methyl-D-aspartate receptor (anti-NMDAR) encephalitis often causes cognitive impairment, yet conventional MRI lacks sensitivity to detect related microstructural changes. This study aimed to investigate T1-weighted/T2-weighted (T1w/T2w) ratios alterations in normal-appearing white matter (NAWM) and their relationship with cognitive function in patients with anti-NMDAR encephalitis, including a 12-month longitudinal follow-up.

**Methods:**

Fifty-nine patients with anti-NMDAR encephalitis (31 with mild cognitive impairment (MCI), 28 with no cognitive impairment (NCI)) and 34 healthy controls (HCs) underwent 3.0T whole-brain MRI and comprehensive neuropsychological assessments, including the Montreal Cognitive Assessment (MoCA), Mini-Mental State Examination (MMSE), and domain-specific tests for attention-executive function, memory, and visuospatial reasoning. T1w/T2w ratios were calculated for globel NAWM and multiple major white matter (WM) tracts. Eighteen MCI patients were followed longitudinally for 12 months to evaluate changes in T1w/T2w ratios and cognitive performance over time.

**Results:**

Compared with NCI patients and HCs, MCI patients exhibited significantly reduced T1w/T2w ratios in global NAWM (*P* < 0.001) and multiple WM tracts, including the corpus callosum, fornix, left hippocampal cingulum, and right hippocampal cingulum (all *P* < 0.05). Lower ratios were significantly associated with worse global cognition (r = 0.364, *P* = 0.023), memory (r = 0.336, *P* = 0.023), and visuospatial reasoning performance (r = 0.397, *P* = 0.005). In the 18 MCI patients with 12-month follow-up, increased T1w/T2w ratios in the fornix and hippocampal cingulum (both *P* = 0.009) were accompanied by significant improvements in visuospatial reasoning and memory functions.

**Conclusion:**

T1w/T2w ratios derived from routine MRI provide a sensitive, clinically feasible biomarker for detecting NAWM microstructural damage in anti-NMDAR encephalitis. Longitudinal changes in these ratios may parallel cognitive improvement, and these preliminary findings support further investigation of this metric as an imaging biomarker, independent cohorts.

## 1 Introduction

Anti-N-methyl-D-aspartate receptor (anti-NMDAR) encephalitis is an autoimmune disorder that has attracted increasing attention in recent years owing to its complex pathophysiology and diverse clinical manifestations, including psychiatric symptoms, seizures, dysphasia, memory loss, and involuntary movements (1, 2). Cognitive impairment represents a core and often debilitating feature of anti-NMDAR encephalitis. Previous studies have shown that cognitive deficits are prevalent among patients even in the chronic phase of the disease. Although some patients exhibit only mild cognitive impairments that may not be readily noticeable in daily life, others experience severe cognitive decline that substantially compromises their social functioning and overall quality of life (3, 4).

Magnetic resonance imaging (MRI) plays a crucial role in the diagnosis of anti-NMDAR encephalitis, but conventional sequences often reveal no abnormalities or only nonspecific cortical hyperintensities on T_2_-fluid-attenuated inversion recovery (T_2_-FLAIR) and T_2_-weighted imaging (T_2_WI), which limits diagnostic specificity (5). Advanced techniques such as diffusion tensor imaging (DTI), resting-state functional MRI (fMRI), and magnetic resonance spectroscopy provide more detailed insights but require specialized equipment, extended scanning times, and complex analytical processes, thereby restricting their routine clinical application (6–8). In contrast, the T1w/T2w ratio can be readily derived from standard MRI sequences, offering a cost-effective and practical tool for clinical monitoring (9–11).

The T1-weighted/T2-weighted (T1w/T2w) ratio, widely applied to detect white matter (WM) damage and normal-appearing white matter (NAWM) in various central nervous system (CNS) disorders, can be obtained from conventional MRI without complex processing pipelines. Prior studies have demonstrated its utility: in multiple sclerosis, it reveals heterogeneous microstructural damage patterns across clinical phenotypes and disability stages (9, 10); in Alzheimer’s disease, altered ratios reflect intracortical myelin content and correlate with accelerated cognitive decline (11). Collectively, these findings underscore the T1w/T2w ratio as a promising imaging biomarker for quantifying microstructural pathology across CNS disorders, with clinical value for tracking disease progression.

In this context, Hartung et al. extended the use of the T1w/T2w ratio to anti-NMDAR encephalitis, demonstrating its effectiveness in differentiating microstructural alterations in both gray matter and WM between patients and healthy controls (HCs) (12). However, the simple dichotomous classification of patients in previous work contrasts with our clinical observations, where routine MRI often lacks sensitivity and the psychiatric symptoms vary considerably.

Despite growing insights into the cognitive deficits associated with anti-NMDAR encephalitis, microstructural alterations in NAWM and their association with cognitive function remain underexplored. Therefore, this study aimed to identify and characterize NAWM microstructural changes within specific WM tracts and to evaluate their relationship with cognitive functions in anti-NMDAR encephalitis. Furthermore, a subset of participants underwent a one-year longitudinal follow-up to tract subclinical microstructural changes and to explore the dynamic interplay between microstructural alterations and cognitive outcomes.

## 2 Methods

### 2.1 Standard protocol approvals, registrations, and patient consents

This study was approved by the Institutional Review Board of the First Affiliated Hospital of Chongqing Medical University and was therefore been performed in accordance with the ethical standards laid down in the 1964 Declaration of Helsinki and its later amendments. Written informed consent was obtained from all participants before MRI scanning.

### 2.2 Participants

Fifty-nine patients with anti-NMDAR encephalitis who had passed the acute stage were recruited from the Department of Neurology at the First Affiliated Hospital of Chongqing Medical University from October 2019 to January 2024. Additionally, 34 HCs, matched for sex, age, and education, were recruited from the community. All participants fulfilled the diagnostic criteria for anti-NMDAR encephalitis recommended by Lancet Neurology (13) in 2016, which required: (1) acute onset in individuals aged 18 years or older; (2) absence of pre-existing disability related to anti-NMDAR encephalitis prior to symptom onset; (3) positive anti-NMDAR antibody results in cerebrospinal fluid (CSF) and/or serum; (4) availability of cognitive assessment data; and (5) reasonable exclusion of alternative diagnoses.

Exclusion criteria were as follows: (1) remaining in the acute phase; (2) presence of other neurological disorders; (3) incomplete imaging data; and (4) concurrent use of medications known to affect WM integrity (e.g., long-term corticosteroids). HCs were required to have no history of neurological or psychiatric disorders, or any other CNS-related organic diseases, and all were right-handed. Due to the observational study approach, no a prior power calculation was performed. For the purposes of this study, the acute phase was defined as within 3 months of symptom onset. Only patients who were beyond 3 months from onset and in clinically stable condition were included in the final analysis (**Table 1**).

**Table 1:** Demographic and neuropsychological variables.

	HCs (n = 34)	MCI (n = 31)	NCI (n = 28)	*P* value
Demographic characteristics				
Age	32.79 ± 7.73	29.77 ± 7.17	33.28 ± 9.66	0.198
sex	24/10	19/12	21/7	0.505^c^
Education	11.59 ± 3.11	10.81 ± 2.80	11.71 ± 3.11	0.601
Acute-phase clinical indicators				
Time to diagnosis (days)	–	20.6 ± 14.0	19.5 ± 17.0	0.346^d^
Time to first immunotherapy (days)	–	16.7 ± 12.1	17.7 ± 17.9	0.715^d^
Length of hospitalization (days)	–	32.8 ± 21.6	27.1 ± 14.2	0.353^d^
Interval from onset to MRI (months)	–	7.5 ± 2.3	7.6 ± 3.1	0.879^d^
Acute-phase immunotherapy (n, %)				
Corticosteroids	–	23/31 (74.2%)	19/28 (67.9%)	0.592^c^
IVIG	–	12/31 (38.7%)	12/28 (42.9%)	0.746^c^
Second-line immunotherapy	–	5/30 (16.7%)	7/28 (25%)	0.465^c^
Acute stage symptoms (n, %)				
Seizures		20/51 (39.2%)	13/51 (25.5%)	0.063^c^
Psychiatric symptoms	–	19/55 (34.5%)	19/55 (34.5%)	0.311^c^
Impaired consciousness	–	13/51 (25.5%)	19/51 (37.3%)	0.120^c^
Movement disorder	–	14/54 (25.9%)	16/54 (29.6%)	0.246^c^
Autonomic dysfunction	–	6/26 (23.1%)	10/26 (38.5%)	0.263^c^
Neuropsychological tests				
MoCA	28.18 ± 1.27	19.59 ± 4.19	26.96 ± 2.33	< 0.001
MMSE	27.97 ± 1.03	25.48 ± 1.43	26.71 ± 1.51	< 0.001
Attention-execution	-0.01 ± 0.59	-1.54 ± 1.39	-0.45 ± 0.93	< 0.001
Memory	-0.01 ± 0.47	-1.63 ± 0.61	-0.71 ± 0.59	< 0.001
Visuospatial reasoning	0.00 ± 1.00	-1.75 ± 0.71	-0.44 ± 0.98	< 0.001^a^
Characteristics	HCs vs MCI *P* value	HCs vs NCI *P* value	NCI vs MCI *P* value	
Age	0.211	0.815	0.310	
sex ^c^	0.429^c^	0.698^c^	0.260	
Education	0.424	0.889	0.350	
MoCA	< 0.001	0.030	< 0.001	
MMSE	< 0.001	0.001	0.002	
Attention-execution^a^	< 0.001	0.056	0.007	
Memory	< 0.001	< 0.001	< 0.001	
Visuospatial reasoning^a^	< 0.001	0.047	< 0.001	

MCI, mild cognitive impairment; NCI, no cognitive impairment; HCs, healthy controls; MoCA, Montreal Cognitive Assessment; MMSE, Mini-Mental State Examination. All comparisons remained significant after FDR correction unless otherwise stated.

Values are presented as the mean ± standard deviation, except for sex distribution.

^a^ Unless otherwise indicated, P values were calculated with one-way ANOVA test among three groups.

^b^ Unless otherwise indicated, P values were calculated with two-tailed t-tests.

^c^ P Value was obtained using chi-squared tests.

^d^ P Value was obtained using Mann-Whitney U test.

### 2.3 Cognitive assessment

All participants underwent a comprehensive battery of cognitive assessments administered by two experienced neurologists within one week after MRI scanning. The Montreal Cognitive Assessment (MoCA) and the Mini-Mental State Examination (MMSE) were utilized to evaluate overall cognitive performance. Z-scores were calculated for three cognitive domains (attention-executive function, memory function, and visuospatial reasoning), constructed from 5 subdomains and were adjusted for age, sex, and years of education.

Processing speed was assessed using the Symbol Digit Modalities Test, and executive functions using the Category Verbal Fluency Test; both are subdomains of attention-executive functions (3). Working memory was assessed using the Digit Span Test, and episodic memory was evaluated using the Rey Auditory Verbal Learning Test (including both short- and long-delay recall); both are subdomains of the memory domain. Fluid intelligence (visuospatial reasoning) was assessed with Raven’s Progressive Matrices.

Cognitive performance was considered impaired if the score was ≤ 1.5 standard deviations below the mean of the HCs group, adjusted for age, sex, and years of education. Impairments in three or four cognitive domains were defined as MCI (a study-specific classification, not a clinical diagnosis), whereas impairments in fewer than three domains were defined as NCI (3). Accordingly, thirty-one patients were classified into the MCI group and twenty-eight into the NCI group.

### 2.4 Longitudinal follow-up

To further examine the relationship between subclinical white matter microstructural evolution and cognitive function, we conducted a one-year longitudinal study involving eighteen patients with MCI. This sample size was determined by the number of patients who completed the scheduled follow-up assessments, as some patients were lost to follow-up or were unable to return for repeat evaluations due to limited compliance and scheduling constraints. In this study, we hypothesized that T1w/T2w ratios could reflect subclinical microstructural changes rather than clinical disease progression. As a pre-registered secondary analysis, we investigated whether microstructural changes in specific WM tracts (identified in prior cross-sectional analysis) correlate with domain-specific cognitive improvements. Specifically, 12 months after the baseline MRI scan and cognitive assessment, the same procedures were performed in these eighteen patients. All patients had received baseline immunotherapy and remained clinically stable throughout the observation period, without recurrence.

### 2.5 MRI acquisition

MRI investigations were performed on a 3.0-T scanner (Magnetom Skyra, Siemens Healthcare GmbH, Erlangen, Germany) using a 32-channel head coil. The following sequences were acquired:

- Sagittal 3D T_1_-weighted magnetization prepared rapid gradient echo (MPRAGE): repetition time (TR) = 2,300 ms, echo time (TE) = 2.26 ms, voxel size = 1 × 1 × 1 mm^3^, field of view (FOV) = 256 × 256 mm^2^, slices = 192, inversion time = 900 ms.
- Sagittal 3D T_2_-weighted SPACE: TR = 3,000 ms, TE = 380 ms, FOV = 256 × 256 mm^2^, voxel size = 1 × 1 × 1 mm^3^, slices = 176.
- Sagittal 3D FLAIR: TR = 5,000 ms, TE = 388 ms, inversion time = 1800 ms, FOV = 256 × 256 mm^2^, voxel size = 0.5 × 0.5 × 1 mm^3^, slices = 192.

No technical modifications were made to the MRI scanner or imaging parameters between 2019 and 2024.

### 2.6 MRI data processing and analysis

**Figure 1:**
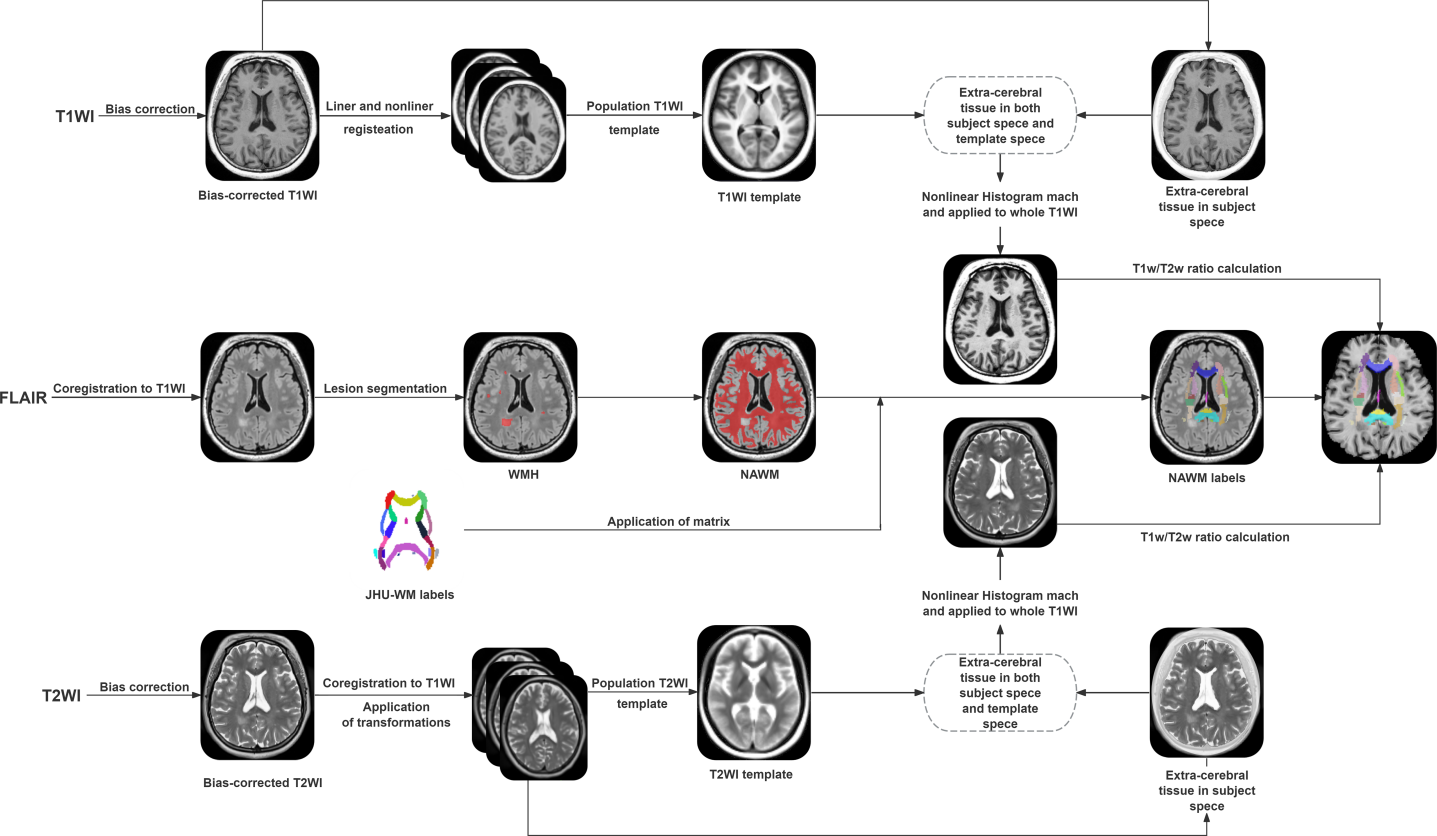
Workflow of data post-processing in this study. FLAIR, fluid-attenuated inversion recovery; WMH, white matter hyperintense; NAWM, normal appearing white matter; T1w/T2w ratio, T1-weighted/T2-weighted ratio.

The data processing workflow is illustrated in **Figure 1** and includes four main stages: bias correction, registration, segmentation, and nonlinear histogram calibration followed by T1w/T2w ratio calculation.

First, bias-field correction was applied to T_1_-weighted, T_2_-weighted, and FLAIR images using Statistical Parametric Mapping (SPM) software (14). Second, the Computational Anatomy Toolbox (CAT12, v.11.0938) in SPM12 (15) was used to generate a brain mask and segment tissue into WM and CSF. The T_1_-weighted, T_2_-weighted, and FLAIR images were then rigidly registered to the individual T_1_-weighted image using FMRIB’s Linear Image Registration Tool (FLIRT). Subsequently, non-linear registration with FSL (https://fsl.fmrib.ox.ac.uk/fsl/) (16) was performed to align the images to the standard Montreal Neurological Institute (MNI) space.

Lesion masks were extracted from T_2_-weighted and FLAIR images using the Lesion Segmentation Tool (LST) and SPM12 (17), after coregistration to the individual T_1_-weighted image (T_1_WI). For accuracy, two experienced researchers and one neurologist manually corrected the WM lesion masks using the ITK-SNAP 3.8.0 (accessed in June 2019) (18). NAWM was defined as WM excluding lesion areas. To investigate the relationship between the T1w/T2w ratios and cognition, regions of interest (ROIs) were extracted from WM tracts and mapped from MNI space to individual T_1_-weighted space using inverse deformation fields. ROIs were derived from the Johns Hopkins University (JHU) WM atlas, which contains 48 tracts (available at https://identifiers.org/neurovault.collection:264).

T1w/T2w ratio maps were generated following the procedure described by Cappelle et al. (19). An average template of uncalibrated T_1_-weighted and T_2_-weighted images from all 93 subjects in MNI space was first computed. Intensity histograms of extracerebral tissue were then extracted from both the individual images and the template. A nonlinear histogram matching procedure, implemented in the MRtrix3 package (http://www.mrtrix.org/) (20), was applied to each subject’s T_1_-weighted image to produce calibrated images, and the same procedure was applied to T_2_-weighted images. The final T1w/T2w ratio map was obtained by dividing the calibrated T_1_-weighted images by the calibrated T_2_-weighted image.

### 2.7 Statistical analysis

Statistical analyses were performed using SPSS software (IBM SPSS Statistics 23.0; IBM, Armonk, NY, USA). Prior to inter-group comparisons, the Kolmogorov-Smirnov test was used to assess the normality of the data. The chi-square test was applied to compare sex distribution between patients and the HCs group. Age, years of education, and cognitive scores were compared using the Kruskal-Wallis H test and Mann-Whitney U tests for non-parametric data, while one-way analysis of variance (ANOVA) was used for normally distributed data.

For multiple group comparisons, an analysis of covariance (ANCOVA) was performed, followed by *post-hoc* t-tests, to compare T1w/T2w ratios among individuals with MCI, NCI, and HCs, with age, sex, and education level as covariates. For measures that showed significant differences in the ANCOVA, partial Spearman correlation analysis was conducted to assess the relationship between T1w/T2w ratios and cognitive scores, controlling for age, sex, and education.

For the longitudinal study, the Wilcoxon signed-rank test and paired t-test were used to examine changes in cognitive performance and subregional T1w/T2w ratio alterations. The linear mixed-effect (LME) model, implemented in R software (v. 4.4.3; lme4 package v.1.1.36), was used to investigate whether the T1w/T2w ratios in NAWM moderated longitudinal changes in cognitive function over time. In this model, MMSE, MoCA, attention-executive function scores, memory scores, and visuospatial reasoning scores were considered as outcome variables. Sex, age, education level, and subregion T1w/T2w ratios × time were treated as independent variables. The interaction terms T1w/T2w ratios × time was the effect of interest, as it reflected whether T1w/T2w ratios moderated the relationship between time and cognitive performance. False discovery rate (FDR) correction was applied separately to distinct families of tests (1): all *post-hoc* pairwise comparisons of T1w/T2w ratios among groups; (2) all partial correlation analyses between microstructure and cognitive scores; (3) all longitudinal paired comparisons; and (4) all linear mixed-effect models and interaction analyses. A significance level of *P* < 0.05 was applied throughout, and a corrected threshold of *q* < 0.05 was used for FDR analyses.

## 3 Results

### 3.1 Demographic and clinical characteristics

In this study, 59 patients with anti-NMDAR encephalitis (28 with NCI and 31 with MCI) and 34 HCs from a single cohort were included. The demographic characteristics and cognitive assessment scores of the three groups are summarized in **Table 1**. No significant differences in age, sex distribution, or educational level were observed among the groups (all *P* > 0.1). A significant main effect of group was found for both MoCA and MMSE scores (both *P* < 0.001). Specifically, global cognitive performance was significantly lower in the MCI group compared with the NCI and HCs groups (both *P* < 0.001).

### 3.2 Group comparisons of T1w/T2w ratios

**Figure 2:**
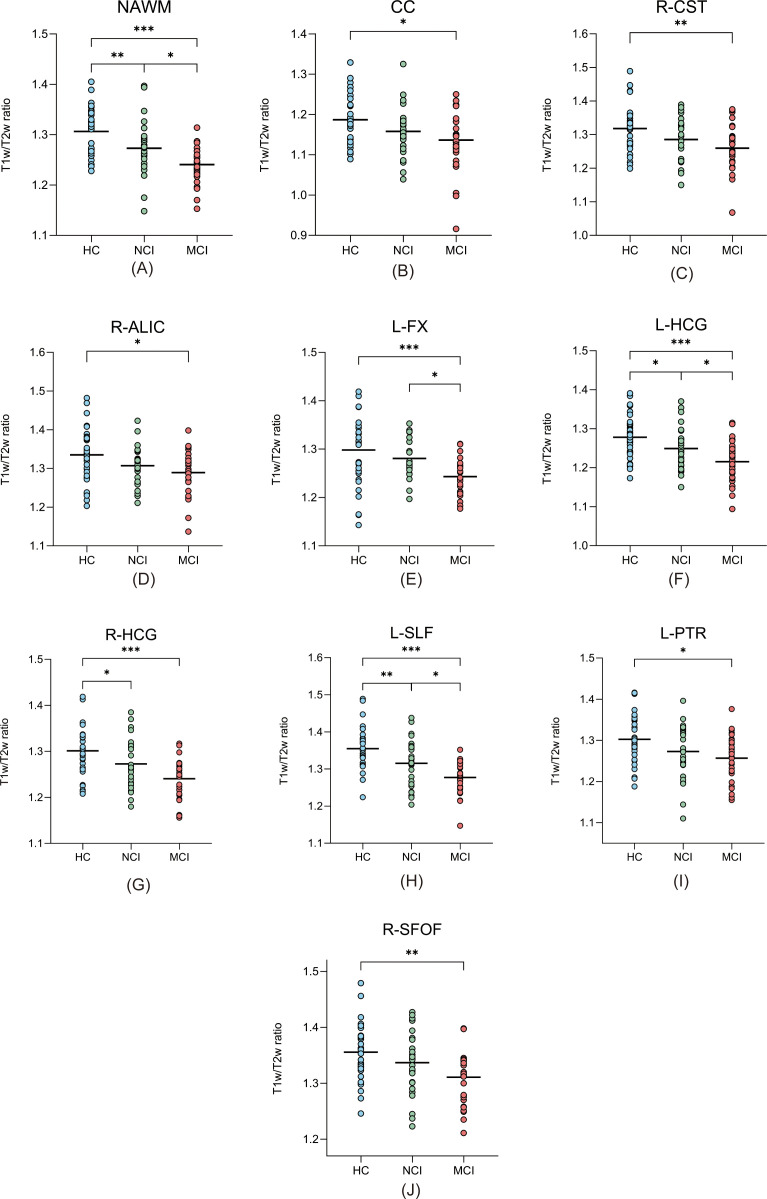
Group comparisons of T1w/T2w ratios in NAWM **(A)** and specific WM tracts **(B–J)** among the MCI, NCI, and HCs groups. Statistical significance is indicated by asterisks (*** P < 0.001; ** P < 0.01; *P < 0.05). All comparisons remained significant after FDR correction unless otherwise stated. NAWM, normal-appearing white matter; MCI, mild cognitive impairment; NCI, no cognitive impairment; HCs, healthy controls; CC, corpus callosum; CST, corticospinal tract; ALIC, anterior limb of internal capsule; FX, fornix; HCG, hippocampal cingulum; SLF, superior longitudinal fasciculus; PTR, posterior thalamic radiation; SFOF, superior fronto-occipital fasciculus.

**Figure 3:**
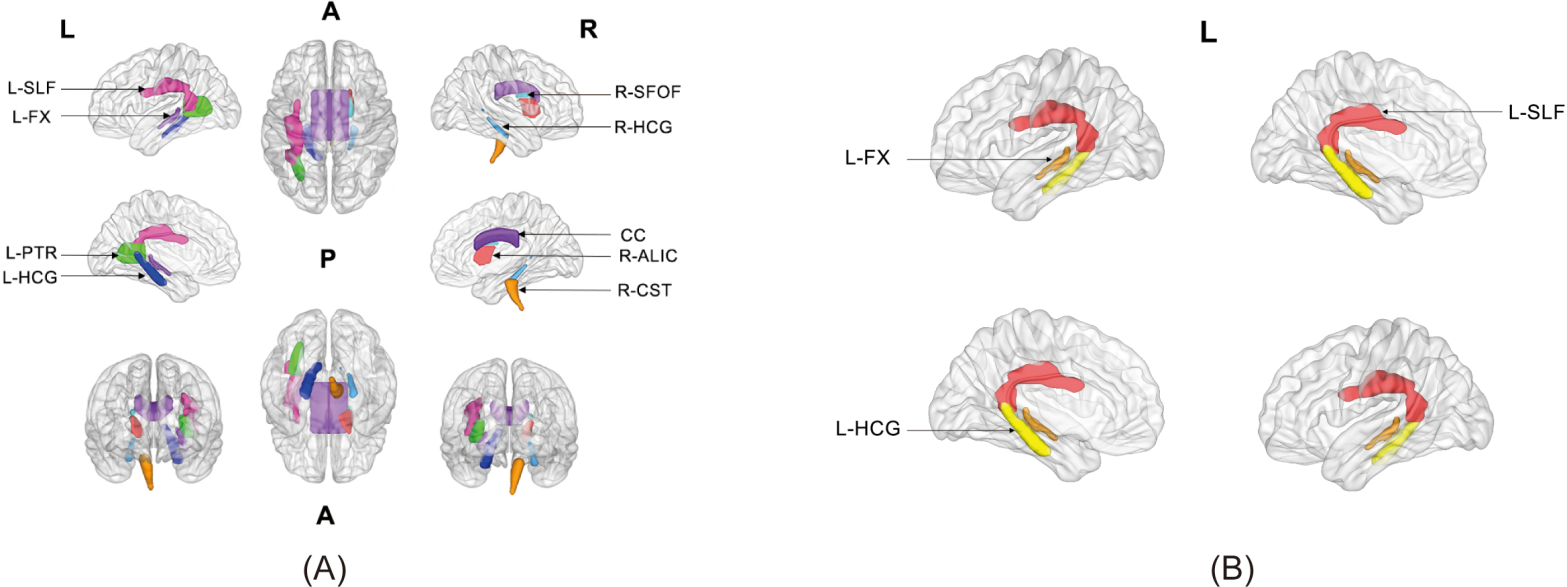
3D illustration of parcellations showing significant differences in T1w/T2w ratios between the HCs and MCI groups **(A)**, and between the MCI and NCI groups **(B)**. All comparisons remained significant after FDR correction unless otherwise stated. MCI, mild cognitive impairment; NCI, no cognitive impairment; HCs, healthy controls; CC, corpus callosum; CST, corticospinal tract; ALIC, anterior limb of internal capsule; FX, fornix; HCG, hippocampal cingulum; SLF, superior longitudinal fasciculus; PTR, posterior thalamic radiation; SF-OF, superior fronto-occipital fasciculus; L, left; R, right; A, anterior; P, posterior.

Comparisons of T1w/T2w ratios in NAWM among the MCI, NCI, and HCs groups are summarized in **Table 2** and illustrated in **Figure 2**. A significant group effect was observed for NAWM T1w/T2w ratios (*P* < 0.001, FDR-corrected). In addition, significant differences were found in several WM tracts, including the corpus callosum, right corticospinal tract, right anterior limb of the internal capsule, left fornix, bilateral hippocampal cingulum, left superior longitudinal fasciculus, left posterior thalamic radiation, and right superior fronto-occipital fasciculus (all *P* < 0.05, FDR-corrected). Subregions of WM with significant differences are illustrated in a 3D representation (21) (**Figure 3**).

**Table 2:** Results of T1w/T2w ratios in the NAWM and several specific white matter tracts.

White matter regions	HCs (n = 34)	MCI (n = 31)	NCI (n = 28)	*P* value^a^
NAWM	1.31 ± 0.05	1.24 ± 0.03	1.27 ± 0.05	< 0.001
Corpus callosum	1.19 ± 0.06	1.14 ± 0.07	1.16 ± 0.06	0.023
R-corticospinal tract	1.32 ± 0.07	1.26 ± 0.07	1.29 ± 0.06	0.009
R-anterior limb of internal capsule	1.33 ± 0.07	1.29 ± 0.06	1.31 ± 0.05	0.027
L-fornix	1.30 ± 0.07	1.24 ± 0.03	1.28 ± 0.04	0.001
L-hippocampal cingulum	1.28 ± 0.05	1.22 ± 0.05	1.25 ± 0.06	< 0.001
R-hippocampal cingulum	1.30 ± 0.06	1.24 ± 0.04	1.27 ± 0.05	< 0.001
L-superior longitudinal fasciculus	1.35 ± 0.06	1.28 ± 0.04	1.32 ± 0.06	< 0.001
L-posterior thalamic radiation	1.30 ± 0.06	1.26 ± 0.05	1.27 ± 0.07	0.015
R-superior fronto-occipital fasciculus	1.36 ± 0.05	1.31 ± 0.05	1.34 ± 0.06	0.012	
White matter regions	HCs vs MCI *P* value^b^	HCs vs NCI *P* value^b^	NCI vs MCI *P* value^b^	
NAWM	< 0.001	0.007	0.027	
Corpus callosum	0.024	0.104	0.411	
R-corticospinal tract	0.009	0.058	0.385	
R-anterior limb of internal capsule	0.034	0.075	0.581	
L-fornix	0.001	0.165	0.040	
L-hippocampal cingulum	< 0.001	0.047	0.05	
R-hippocampal cingulum	< 0.001	0.036	0.060	
L-superior longitudinal fasciculus	< 0.001	0.007	0.031	
L-posterior thalamic radiation	0.017	0.063	0.472	
R-superior fronto-occipital fasciculus	0.009	0.145	0.210	

NAWM, normal-appearing white matter; MCI, mild cognitive impairment; NCI, no cognitive impairment; HCs, healthy controls; L, left; R, right. All comparisons remained significant after FDR correction unless otherwise stated.

^a^ P value of the cross-sectional group comparisons (ANCOVAs with age, sex, and education as covariates; FDR corrected).

^b^ P value of *post-hoc* comparisons (FDR corrected).

Results are presented as mean ± standard deviation.

In pairwise comparisons, MCI patients showed significantly reduced T1w/T2w ratios compared with HCs in multiple brain regions, including the NAWM, corpus callosum, right corticospinal tract, right anterior limb of the internal capsule, left fornix, bilateral hippocampal cingulum, left superior longitudinal fasciculus, left posterior thalamic radiation, and right superior fronto-occipital fasciculus (all *P* < 0.05, FDR-corrected). Moreover, significant reductions were observed in the MCI group compared with the NCI group in NAWM, the left fornix, left hippocampal cingulum, and left superior longitudinal fasciculus (all *P* < 0.05, FDR-corrected).

### 3.3 Associations with cognitive performance

**Figure 4:**
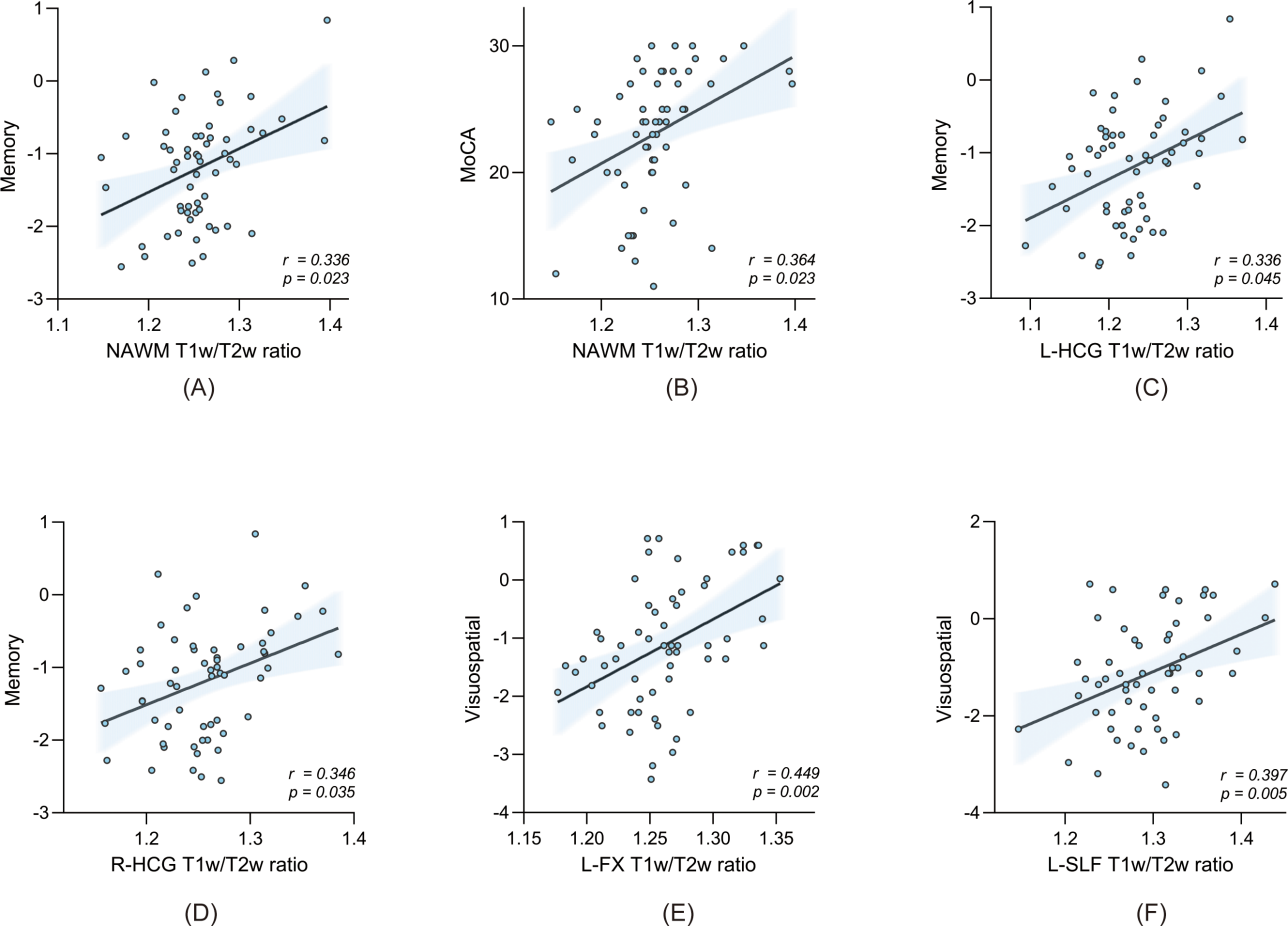
Scatterplot illustrating the relationships between WM T1w/T2w ratios and general cognitive scores **(A)**, as well as domain-specific cognitive z-scores **(B–F)**, in all participants. A fitted line is shown when a significant partial correlation exists, controlling for age, sex, and education. All correlations remained significant after FDR correction unless otherwise stated. NAWM, normal-appearing white matter; FX, fornix; HCG, hippocampal cingulum; SLF, superior longitudinal fasciculus; MoCA, Montreal Cognitive Assessment; MMSE, Mini-Mental State Examination; L, left; R, right.

Partial Spearman correlation analyses in MCI and NCI groups, controlling for age, sex, and education, revealed significant associations between T1w/T2w ratios in specific WM tracts and cognitive performance (**Table 3**; **Figure 4**). The T1w/T2w ratio in NAWM was positively correlated with global cognitive function assessed by MoCA (*r* = 0.364, *P* = 0.023), and with memory function (*r* = 0.336, *P* = 0.023).

**Table 3:** Partial Spearman correlations between some T1w/T2w ratios parameters and cognitive rating scales.

White matter regions	MoCA	MMSE	Attention- execution	Memory	Visuospatial reasoning
NAWM	*r* = 0.364 *P* = 0.023	*r* = 0.171 *P* = 0.195	*r* = 0.055 *P* = 0.681	*r* = 0.336^*^ P = 0.023	*r* = 0.250 *P* = 0.056
Corpus callosum	*r* = 0.121 *P* = 0.361	*r* = 0.071 *P* = 0.592	*r* = -0.006 *P* = 0.961	*r* = 0.01 *P* = 0.995	*r* = 0.130 *P* = 0.325
R-corticospinal tract	*r* = 0.068 *P* = 0.607	*r* = -0.166 *P* = 0.210	*r* = -0.069 *P* = 0.604	*r* = 0.006 *P* = 0.966	*r* = 0.103 *P* = 0.436
R-anterior limb of internal capsule	*r* = 0.016 *P* = 0.904	*r* = 0.085 *P* = 0.521	*r* = -0.001 *P* = 0.995	*r* = 0.055 *P* = 0.681	*r* = 0.133 *P* = 0.316
L-fornix	*r* = 0.317^*^ P = 0.035	*r* = 0.165 *P* = 0.212	*r* = 0.092 *P* = 0.489	*r* = 0.210 *P* = 0.183	*r* = 0.449^**^ P = 0.002
L-hippocampal cingulum	*r* = 0.228 *P* = 0.083	*r* = 0.140 *P* = 0.291	*r* = 0.055 *P* = 0.677	*r* = 0.336^*^ P = 0.045	*r* = 0.115 *P* = 0.385
R-hippocampal cingulum	*r* = 0.272 *P* = 0.062	*r* = 0.149 *P* = 0.259	*r* = 0.083 *P* = 0.530	*r* = 0.346^*^ P = 0.035	*r* = 0.284 *P* = 0.062
L-superior longitudinal fasciculus	*r* = 0.390^**^P = 0.005	*r* = 0.180 *P* = 0.173	*r* = 0.075 *P* = 0.573	*r* = 0.216 *P* = 0.100	*r* = 0.397^**^ P = 0.005
L-posterior thalamic radiation	*r* = 0.161 *P* = 0.223	*r* = 0.065 *P* = 0.624	*r* = 0.187 *P* = 0.157	*r* = 0.141 *P* = 0.286	*r* = 0.195 *P* = 0.139
R-superior fronto-occipital fasciculus	*r* = 0.294 *P* = 0.120	*r* = 0.05 *P* = 0.970	*r* = 0.256 *P* = 0.051	*r* = 0.212 *P* = 0.106	*r* = 0.161 *P* = 0.223

Analysis for the general cognitive scores included data from patients (n = 59). All correlations remained significant after FDR correction unless otherwise specified.

NAWM, normal-appearing white matter; MoCA, Montreal Cognitive Assessment; MMSE, Mini-Mental State Examination.

Partial Spearman correlations, controlling for age, sex, and education, are used to assess the associations between T1w/T2w ratios and cognitive performance in all participants (**P < 0.01; *P < 0.05).

Among the specific WM tracts, the left fornix showed significant positive correlations with both MoCA (*r* = 0.317, *P* = 0.035) and visuospatial reasoning (*r* = 0.449, *P* = 0.002). Bilateral hippocampal cingulum were positively correlated with memory function (left: *r* = 0.336, *P* = 0.045; right: *r* = 0.346, *P* = 0.035). In addition, T1w/T2w ratios in the left superior longitudinal fasciculus were positively correlated with MoCA (*r* = 0.390, *P* = 0.005) and visuospatial reasoning (*r* = 0.397, *P* = 0.005). All results remained significant after FDR correction.

### 3.4 Longitudinal changes in cognition and WM microstructure

**Figure 5:**
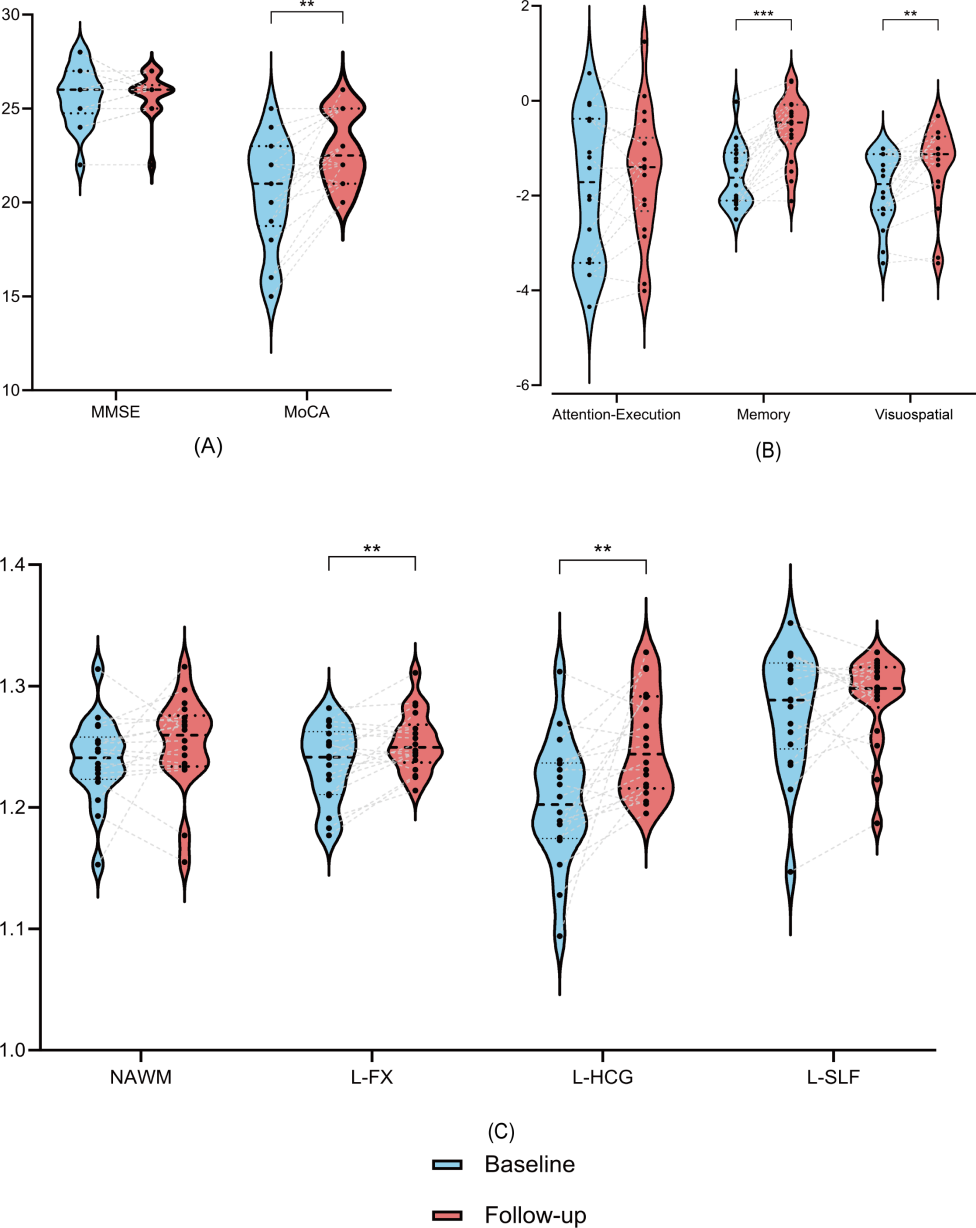
Longitudinal changes from baseline (blue) to follow-up (red) in cognitive performance **(A, B)** and T1w/T2w ratios in NAWM and subregions **(C)**. All comparisons remained significant after FDR correction unless otherwise stated. Statistical significance is indicated by asterisks (***P < 0.001; **P < 0.01). MoCA, Montreal Cognitive Assessment, MMSE, Mini-Mental State Examination, NAWM, normal-appearing white matter; LFX, left fornix; LHCG, left hippocampal cingulum; LSLF, left superior longitudinal fasciculus.

Improvements from the baseline and follow-up MRI scans were observed across nearly all cognition domains (**Table 4**; **Figure 5**). In particular, memory function showed significant gains, as reflected by better performance on the Digit Span Test and Rey Auditory Verbal Learning Test (*P* < 0.001). Significant improvements were also noted in MoCA (*P* = 0.006) and visuospatial reasoning (*P* = 0.009) at the 12-month follow-up. Furthermore, significant increases in T1w/T2w ratios were observed in the left fornix and left hippocampal cingulum (all *P* < 0.05, **Table 4**).

**Table 4:** Baseline and follow-up T1w/T2w ratios in NAWM and some subregions, as well as cognitive progression.

Measurements	Baseline	12 months follow-up	*P* value
MoCA	19.06 ± 4.12	22.83 ± 1.92	0.006^b^
MMSE	25.61 ± 1.54	25.78 ± 1.17	0.523^b^
Attention-execution	-1.84 ± 1.55	-1.54 ± 1.33	0.300^a^
Memory	-1.58 ± 0.65	-0.59 ± 0.70	< 0.001^a^
Visuospatial reasoning	-1.93 ± 0.74	-1.35 ± 0.89	0.009^b^
NAWM	1.24 ± 0.03	1.25 ± 0.04	0.303^a^
Left fornix	1.24 ± 0.03	1.25 ± 0.02	0.009^a^
L-hippocampal cingulum	1.20 ± 0.05	1.25 ± 0.04	0.009^a^
L-superior longitudinal fasciculus	1.28 ± 0.05	1.29 ± 0.04	0.356^b^

MoCA, Montreal Cognitive Assessment; MMSE, Mini-Mental State Examination; NAWM, normal-appearing white matter. All *P* values after FDR correction.

^a^ Paired t-test and ^b^Wilcoxon signed-rank test are used to evaluate changes in cognitive performance and alterations in subregional T1w/T2w ratios.

In the pre-specified LME model for visuospatial reasoning, a marginally significant interaction was found between the T1w/T2w ratio of the left fornix and time (std *β* = 0.38, uncorrected *P* = 0.028, FDR-corrected *P* = 0.112). Including the time × left fornix T1w/T2w ratio interaction term improved model fit (model with interaction, Akaike’s information criterion (AIC) = 81.90, Bayesian information criterion (BIC) = 96.15; model without interaction term, AIC = 90.06, BIC = 102.73). In the memory function model, the interaction between the T1w/T2w ratio of the left hippocampal cingulate and time also reached marginally statistical significance for memory function (std *β* = 0.32, uncorrected *P* = 0.066).

### 3.5 Confounding factor analysis

Confounding factor analyses were conducted to assess the potential influence of key demographic and clinical factors on the observed white matter microstructural measures. The T1w/T2w ratios in the regions of interest showed no significant association with the presence of specific acute-phase clinical symptoms, such as seizures, psychiatric features, or movement disorders, after correcting for age, sex, and educational level (all *P* values > 0.05, **Supplementary Table S1**). Furthermore, no significant differences in T1w/T2w ratios were found between patient subgroups who received different acute-phase immunotherapy regimens (all *P* values > 0.05, **Supplementary Table S2**). Analyses of disease time-course variables, including the interval from symptom onset to MRI scan, also revealed no significant correlations with white matter integrity measures (all *P* values > 0.05, **Supplementary Table S3**).

## 4 Discussion

In this study, we observed significant alterations in T1w/T2w ratios within NAWM and several major WM tracts, including the corpus callosum, corticospinal tract, anterior limb of the internal capsule, fornix, hippocampal cingulum, superior longitudinal fasciculus, posterior thalamic radiation, and superior fronto-occipital fasciculus. These alterations were significantly associated with overall and domain-specific cognitive performance, and longitudinal analysis further demonstrated that increases in T1w/T2w ratios in certain tracts may parallel improvements in cognitive function. Collectively, these findings suggest that microstructural abnormalities in NAWM contribute to cognitive impairment in anti-NMDAR encephalitis and that increased T1w/T2w ratios may parallel longitudinal improvements in cognitive function. Together, these methodological advances—specifically, subdividing patients into MCI and NCI groups rather than treating them as a single patient cohort, and incorporating a one-year longitudinal follow-up—enabled us to capture tract-specific microstructural alterations and their dynamic relationship with cognitive recovery. This extends previous cross-sectional work (12) and enhances the translational relevance of T1w/T2w imaging as a clinically feasible biomarker.

Conventional MRI often revealed minimal abnormalities in patients with anti-NMDAR encephalitis. In our cohort, only 19 of 59 patients showed visible lesions on T_2_WI and FLAIR, and these lesions were small and unrelated to the observed clinical symptoms. Importantly, a normal MRI did not preclude severe disease progression or poor long-term outcomes (22). Indeed, multiple studies have reported no correlation between abnormal findings on routine MRI and the long-term prognosis in patients with anti-NMDAR encephalitis (23–26). Thus, the visible lesions may represent only the “tip of the iceberg” of the underlying pathology. These subtle findings further suggest that extensive damage to NAWM could be a crucial yet frequently overlooked component in the pathogenesis of anti-NMDAR encephalitis (27, 28).

Previous studies have confirmed the potential of the T1w/T2w ratio as a biomarker for anti-NMDAR encephalitis (12). In our study, we utilized extracerebral masks derived from T_1_-weighted and T_2_-weighted templates generated from our research population to perform non-linear calibration of the participants’ T_1_- and T2-weighted images. Cappelle et al. demonstrated that this calibration method effectively reduces data variability and minimizes errors related to factors such as scan timing, thereby enhancing inter-subject comparability (19).

In our study, we observed a progressive decrease in T1w/T2w ratios in WM from HCs to the MCI group. This trend was evident in both global NAWM and its subregions. In light of previous post-mortem and basic research, such a pattern may be associated with reduced WM integrity in patients with anti-NMDAR encephalitis. Anti-NMDAR antibodies trigger receptor internalization and neuronal dysfunction, while the infiltration of diverse immune cells creates a pro-inflammatory microenvironment that damages oligodendrocytes. Crucially, the resulting neuronal NMDAR hypofunction disrupts glutamate signaling required for oligodendrocyte-mediated myelination (29, 30). The reduced T1w/T2w ratio in NAWM of MCI patients likely indicates persistent WM microstructural damage and impaired NMDAR function in oligodendrocytes, ultimately exerting detrimental effects on myelination and remyelination.

However, it is important to note that the T1w/T2w ratio is a non-specific imaging marker; while decreased ratios often correlate with demyelination, they may also be influenced by concurrent inflammatory edema, gliosis, or axonal loss. In the recovery/chronic phase of encephalitis, persistent demyelination likely plays a primary role, but potential contributions from residual inflammation cannot be excluded. These findings may reflect immune-mediated inflammation targeting WM structures.

The pattern of white matter damage may bear important implications for functional and cognitive outcomes. Among WM tracts, the hippocampal cingulum plays a crucial role in the limbic system, facilitating information transmission between the hippocampus, frontal lobe, and parietal lobe (31, 32). The preferential involvement of the hippocampal cingulum may reflect the high density of NMDARs in hippocampal neurons, making their projecting fibers susceptible to antibody-mediated demyelination. Our study revealed decreased T1w/T2w ratios in the bilateral hippocampal cingulum of MCI patients, possibly due to the high NMDAR density in the hippocampus, which makes it a primary target for autoantibody attacks (33, 34). Furthermore, significant differences in T1w/T2w ratios in the left hippocampal cingulum were observed between MCI and NCI groups. Complementing this cross-sectional observation, a marginally significant time-dependent interaction was identified between increases in T1w/T2w ratios within the hippocampal cingulum and memory improvement in longitudinal assessments. This dual-phase evidence supports the potential of this microstructural metric to reflect subclinical microstructural changes associated with cognitive recovery, although the marginal statistical significance after FDR correction highlights the need for larger longitudinal studies.

Consistent with our findings, previous literature has reported that WM integrity within the hippocampal cingulum correlates with impaired episodic memory and general cognitive function (29, 35, 36). The hippocampal cingulum, therefore, may serve as a sensitive imaging target to reflect microstructural damage associated with cognitive impairment in anti-NMDAR encephalitis, and its sensitivity to abnormalities suggests the potential of T1w/T2w ratios as a biomarker for assessing disease-related microstructural changes—rather than an indicator for individualized therapy, which requires further validation in dedicated therapeutic studies. Similarly, considering the crucial role of the superior longitudinal fasciculus in visuospatial reasoning, the observed correlation with such deficits is highly plausible (12, 37, 38).

Notably, a predominantly left-hemispheric lateralization of white matter alterations was observed in our study, with significant T1w/T2w ratio reductions detected in the left superior longitudinal fasciculus, left hippocampal cingulum, and left fornix. This lateralization pattern may be attributed to the distinct clinical phenotype of anti-NMDAR encephalitis, which frequently manifests with language deficits (e.g., dysphasia) and psychiatric symptoms. Given that language processing and attention-executive functions are predominantly subserved by left-hemisphere networks, these tracts might be particularly vulnerable to immune-mediated damage or may exhibit more pronounced microstructural changes in response to the disease pathology affecting these specific cognitive domains.

However, because complete seizure data were available for only a subset of 51 patients, we performed a subgroup analysis to explore potential confounding effects. Although no significant differences in white matter T1w/T2w ratios were found between patients with and without acute-phase seizures (**Supplementary Table S1**), the limited sample and incomplete records restrict the strength of this analysis. In our study, although a subgroup analysis did not show significant differences in T1w/T2w ratios between patients with and without acute-phase seizures, we acknowledge that seizure frequency, duration, and subtype (e.g., status epilepticus) could have variable effects on white matter microstructure (39–41). Future studies with detailed seizure quantification and long-term EEG monitoring are warranted to control for this confounding factor more rigorously.

In the longitudinal cohort, 18 MCI patients with acute-phase seizures achieved clinical remission following 12 months of standardized antiepileptic and immunotherapy. Longitudinal analysis demonstrated marginally significant temporal interactions between the left fornical T1w/T2w ratio and visuospatial reasoning improvement during therapeutic interventions. Previous studies have demonstrated that early immunomodulatory interventions effectively reduce seizure frequency while simultaneously improving cognitive outcomes, suggesting a potential pathophysiological link between epileptogenesis and cognitive impairment (42). The fornix, as the principal WM pathway connecting hippocampal and cortical regions, plays a critical role in visuospatial reasoning by facilitating efficient hippocampal-cortical information transfer. Notably, our data revealed decreased T1w/T2w ratios in the left fornix of MCI patients, which were significantly correlated with visuospatial reasoning. Emerging evidence indicates that microstructural compromise within this pathway may contribute to maladaptive reorganization of epileptic networks, thereby potentiating seizure activity while disrupting physiological cognitive circuitry (42). These mechanistic insights provide a plausible explanation for both the observed intergroup differences in fornical T1w/T2w ratios between the MCI and NCI cohorts and the longitudinal changes associated with cognitive improvement during follow-up. Nonetheless, substantial inter-individual variability is observed during follow-up. This heterogeneity may be attributed to differences in treatment regimens during the chronic phase, such as variations in the use of immunosuppressants or antiepileptic drugs, which could differentially influence both microstructural repair and cognitive performance. However, as quantitative EEG monitoring and seizure frequency records were not system conducted during the chronic phase, it remains challenging to disentangle the specific impact of these therapeutic interventions from the natural disease course. Consequently, these factors limit a direct mechanistic interpretation of our longitudinal findings in relation to fluctuating seizure activity and treatment effects.

Several limitations of this study should be noted. First, the relatively small sample size, especially the relatively small sample size, especially the limited number of longitudinal participants (n = 18), may reduce statistical power and restrict the generalizability of our findings. The 18 patients with longitudinal data were enrolled based on routine clinical follow-up without selection bias, and baseline characteristics were comparable between patients with and without follow-up (**Supplementary Table S4**). Additionally, the small healthy control group may compromise the robustness of the normative z-scores used to define cognitive impairment, highlighting the need for validation in larger normative datasets. Second, the cross-sectional design precludes causal inference regarding the relationship between white matter microstructural changes and cognitive impairment. Third, potential confounders—including genetic predispositions, lifestyle factors, comorbidities, CSF antibody titers, antiepileptic drug effects, antibody dynamics, and acute-phase clinical indicators (time to diagnosis, time to first immunotherapy, and length of hospitalization)—were not fully adjusted for in statistical analyses, which may have biased the findings. Moreover, the Clinical Assessment Scale for Autoimmune Encephalitis (CASE), a validated disease severity metric, was not available in this retrospective cohort. In addition, anxiety and depression were not systematically assessed in this cohort, which may represent additional confounding factors for cognitive performance. Fourth, detailed longitudinal data regarding the use, dosage, and duration of medications—including antiepileptic drugs, corticosteroids, and immunosuppressive agents—were not systematically available for all participants. These treatments may independently influence white matter microstructure and cognitive recovery and represent important unmeasured confounders. Furthermore, complete acute stage symptoms data were available for only a subset of patients, limiting the robustness and interpretability of the corresponding subgroup analysis. Fifth, our MCI classification represents a study-specific stratification (based on impairment in ≥3 cognitive domains) rather than standard clinical MCI criteria; thus, caution is warranted when extrapolating these results to clinical populations. Finally, despite a comprehensive neuropsychological battery, our assessments may not fully capture the complex cognitive deficits in anti-NMDAR encephalitis. Future studies should employ larger, multi-center longitudinal cohorts to evaluate our findings and improve statistical robustness. Importantly, integrating imaging biomarkers with CSF antibody titers may help establish mechanistic pathways linking microstructural damage to cognitive outcomes, thereby offering a more comprehensive understanding of disease progression and treatment response.

## 5 Conclusion

Compared to the anti-NMDAR encephalitis patients with NCI and HCs, patients with MCI exhibited an abnormal distribution of T1w/T2w ratios in NAWM. Moreover, T1w/T2w ratios were significantly associated with cognitive scores in these subregions and with disease progression. Taken together, our findings suggest that T1w/T2w ratios can serve as sensitive and clinically feasible biomarkers for detecting microstructural alterations related to cognition and for monitoring disease course in anti-NMDAR encephalitis.

## Data Availability

The raw data supporting the conclusions of this article will be made available by the authors, without undue reservation.
