# Supplementary material for: Microstructural white matter alterations and cognitive impairment in anti-NMDAR encephalitis: evidence from T1w/T2w ratio analysis
Source: Front Immunol. 2026 Apr 29;17:1796544. doi: 10.3389/fimmu.2026.1796544 (PMC13168168; doi:10.3389/fimmu.2026.1796544)
Supplement: Supplementary file 1 [file Table1.docx]

## Supplementary Information

**Table S1. Differences in white matter T1w/T2w ratio between patients with and without symptoms during the acute stage.**

|  | Seizures | Psychiatric symptoms | Impaired consciousness | Movement disorder | Autonomic dysfunction |
| --- | --- | --- | --- | --- | --- |
| NAWM | *P* = 0.488 | *P* = 0.950 | *P* = 0.792 | *P* = 0.996 | *P* = 0.967 |
| Corpus callosum | *P* = 0.488 | *P* = 0.080 | *P* = 0.792 | *P* = 0.608 | *P* = 0.967 |
| R-corticospinal tract | *P* = 0.488 | *P* = 0.670 | *P* = 0.792 | *P* = 0.608 | *P* = 0.967 |
| R-anterior limb of internal capsule | *P* = 0.526 | *P* = 0.950 | *P* = 0.852 | *P* = 0.827 | *P* = 0.967 |
| L-fornix | *P* = 0.488 | *P* = 0.670 | *P* = 0.852 | *P* = 0.946 | *P* = 0.967 |
| R-hippocampal cingulum | *P* = 0.480 | *P* = 0.950 | *P* = 0.919 | *P* = 0.608 | *P* = 0.967 |
| L-hippocampal cingulum | *P* = 0.488 | *P* = 0.808 | *P* = 0.919 | *P* = 0.608 | *P* = 0.967 |
| L-superior longitudinal fasciculus | *P* = 0.526 | *P* = 0.917 | *P* = 0792 | *P* = 0.608 | *P* = 0.967 |
| L-posterior thalamic radiation | *P* = 0.585 | *P* = 0.808 | *P* = 0.792 | *P* = 0.608 | *P* = 0.967 |
| R-superior fronto-occipital fasciculus | *P* = 0.526 | *P* = 0.950 | *P* = 0.852 | *P* = 0.946 | *P* = 0.967 |

Note: NAWM = normal-appearing white matter. General linear models were performed to compare anti-NMDAR encephalitis patients with and without acute-stage symptoms, adjusting for age, gender and educational level. (all *P* values after FDR correction)

**Table S2. Differences in white matter T1w/T2w ratio between anti-NMDAR encephalitis patients receiving different treatments**

|  | Corticosteroids | IVIG | Second line therapy |
| --- | --- | --- | --- |
| NAWM | *P* = 0.841 | *P* = 0.885 | *P* = 0.979 |
| Corpus callosum | *P* = 0.841 | *P* = 0.885 | *P* = 0.801 |
| R-corticospinal tract | *P* = 0.841 | *P* = 0.885 | *P* = 0.801 |
| R-anterior limb of internal capsule | *P* = 0.841 | *P* = 0.885 | *P* = 0.801 |
| L-fornix | *P* = 0.841 | *P* = 0.987 | *P* = 0.801 |
| R-hippocampal cingulum | *P* = 0.841 | *P* = 0.885 | *P* = 0.801 |
| L-hippocampal cingulum | *P* = 0.841 | *P* = 0.987 | *P* = 0.801 |
| L-superior longitudinal fasciculus | *P* = 0.841 | *P* = 0.987 | *P* = 0.801 |
| L-posterior thalamic radiation | *P* = 0.841 | *P* = 0.885 | *P* = 0.801 |
| R-superior fronto-occipital fasciculus | *P* = 0.18 | *P* = 0.987 | *P* = 0.801 |

Note: NAWM = normal-appearing white matter. General linear models were performed to compare anti-NMDAR encephalitis patients receiving different treatments, adjusting for age, gender and educational level. (all *P* values after FDR correction)

**Table S3. Partial Spearman correlation analyses between time-related clinical variables and T1w/T2w ratio in different brain regions**

|  | Time to diagnosis (days) | Time to first immunotherapy (days) | Length of hospitalization (days) | Interval from onset to MRI (months) |
| --- | --- | --- | --- | --- |
| NAWM | *r* = -0.060  *P* = 0.653 | *r* = -0.020  *P* = 0.882 | *r* = -0.124  *P* = 0.354 | *r* = 0.135  *P* = 0.307 |
| Corpus callosum | *r* = 0.032  *P* = 0.809 | *r* = 0.071  *P* = 0.591 | *r* = -0.062  *P* = 0.642 | *r* = 0.201  *P* = 0.127 |
| R-corticospinal tract | *r* = -0.200  *P* = 0.129 | *r* = -0.161  *P* = 0.223 | *r* = 0.064  *P* = 0.634 | *r* = 0.050  *P* = 0.704 |
| R-anterior limb of internal capsule | *r* = -0.037  *P* = 0.779 | *r* = -0.051  *P* = 0.701 | *r* = 0.228  *P* = 0.086 | *r* = 0.026  *P* = 0.845 |
| L-fornix | *r* = -0.065  *P* = 0.625 | *r* = -0.069  *P* = 0.602 | *r* = -0.093  *P* = 0.486 | *r* = 0.210  *P* = 0.111 |
| R-hippocampal cingulum | *r* = 0.200  *P* = 0.129 | *r* = 0.135  *P* = 0.308 | *r* = -0.151  *P* = 0.257 | *r* = 0.151  *P* = 0.252 |
| L-hippocampal cingulum | *r* = 0.118  *P* = 0.372 | *r* = 0.082  *P* = 0.537 | *r* = -0.115  *P* = 0.389 | *r* = 0.131  *P* = 0.322 |
| L-superior longitudinal fasciculus | *r* = -0.140  *P* = 0.291 | *r* = -0.137  *P* = 0.300 | *r* = -0.162  *P* = 0.225 | *r* = -0.031  *P* = 0.813 |
| L-posterior thalamic radiation | *r* = 0.055  *P* = 0.677 | *r* = 0.065  *P* = 0.624 | *r* = 0.200  *P* = 0.133 | *r* = 0.084  *P* = 0.525 |
| R-superior fronto-occipital fasciculus | *r* = 0.081  *P* = 0.540 | *r* = 0.042  *P* = 0.751 | *r* = 0.171  *P* = 0.200 | *r* = -0.115  *P* = 0.384 |

Note: NAWM = normal-appearing white matter. Partial Spearman correlations, controlling for age, sex, and education, are used to assess the associations between T1w/T2w ratios and cognitive performance in all participants. No FDR correction was applied for multiple comparisons.

**Table S4. Baseline characteristics of participants with and without follow-up**

|  | Follow-up | Loss to follow-up | Uncorrected *P-*values |
| --- | --- | --- | --- |
| Gender | 8/10 | 4/9 | 0.125 |
| Age | 28.33 ± 7.77 | 31.08 ± 6.30 | 0.399^b^ |
| Education | 10.22 ± 2.76 | 11.62 ± 2.75 | 0.132^c^ |
| MoCA | 19.06 ± 4.12 | 20.31 ± 4.35 | 0.421 |
| MMSE | 25.61 ± 1.54 | 25.31 ± 1.32 | 0.400^c^ |
| Attention-execution | -1.84 ± 1.55 | -1.13 ± 1.05 | 0.138^d^ |
| Memory | -1.58 ± 0.65 | -1.71 ± 0.57 | 0.582 |
| Visuospatial reasoning | -1.93 ± 0.74 | -1.49 ± 0.62 | 0.089 |

Note: MoCA = Montreal Cognitive Assessment; MMSE = Mini-Mental State Examination. Baseline characteristics were compared between participants who completed follow-up and those who were lost to follow-up. Continuous variables are presented as mean ± standard deviation, and categorical variables are presented as n/N.

^a^ Unless otherwise indicated, *P* values were calculated with independent samples t-test

^b^ *P* Value was obtained using chi-squared tests.

^c^ *P* Value was obtained using Mann-Whitney U test

^d^ *P* Value was obtained using Welch’ t-test
